# Incremental Impact of [^68^ Ga]Ga-PSMA-11 PET/CT in Primary N and M Staging of Prostate Cancer Prior to Curative-Intent Surgery: a Prospective Clinical Trial in Comparison with mpMRI

**DOI:** 10.1007/s11307-021-01650-9

**Published:** 2021-09-14

**Authors:** Florian Szigeti, Gregor Schweighofer-Zwink, Matthias Meissnitzer, Cornelia Hauser-Kronberger, Wolfgang Hitzl, Thomas Kunit, Rosemarie Forstner, Christian Pirich, Mohsen Beheshti

**Affiliations:** 1 Salzburg University of Applied Sciences, Radiation Technology Degree Program, Salzburg, Austria; 2 Division of Molecular Imaging and Theranostics, Department of Nuclear Medicine and Endocrinology, University Hospital, Paracelsus Medical University, Müllner Hauptstraße 48, 5020 Salzburg, Austria; 3 Department of Radiology, University Hospital, Paracelsus Medical University, Salzburg, Austria; 4 Department of Pathology, University Hospital, Paracelsus Medical University, Salzburg, Austria; 5 Research Office (Biostatistics), Paracelsus Medical University, 5020 Salzburg, Austria; 6 Department of Ophthalmology and Optometry, University Hospital, Paracelsus Medical University, 5020 Salzburg, Austria; 7 Research Program Experimental Ophthalmology and Glaucoma Research, Paracelsus Medical University, 5020 Salzburg, Austria; 8 Department of Urology and Andrology, University Hospital, Paracelsus Medical University, Salzburg, Austria

**Keywords:** Prostate cancer, Primary staging, [^68^ Ga]Ga-PSMA-11, PET/CT, Multiparametric MRI

## Abstract

**Purpose:**

The main objective of this prospective study was to assess the value of gallium-68 prostate-specific membrane antigen ([^68^ Ga]Ga-PSMA-11) positron emission tomography/computed tomography (PET/CT) in primary N and M staging of intermediate- and high-risk prostate cancer (PCa) patients before planned curative-intent radical prostatectomy (RPE) and extended pelvic lymph node dissection (ePLND). The second objective was to compare the [^68^ Ga]Ga-PSMA-11 PET/CT findings with standard of care pelvic multi-parametric magnetic resonance imaging (mpMRI) in the detection of locoregional lymph node metastases and intraprostatic prostate cancer.

**Procedures:**

A total of 81 patients (mean age: 64.5 years, baseline mean trigger PSA (tPSA) 15.4 ng/ml, ± 15.9) with biopsy proven PCa (24 intermediate- and 57 high risk) scheduled for RPE and ePLND were enrolled in this prospective study. In 52 patients [^68^ Ga]Ga-PSMA-11 PET/CT, pelvic mpMRI, and RPE with ePLND have been performed. Clinical risk stratification and related biomarkers as well as Gleason score (GS) were recorded. The location of the index lesion (IL) was documented systematically for each modality using a standardized segmentation of the prostate in six segments. Distant bone and lymph node metastasis detected by [^68^ Ga]Ga-PSMA-11 PET/CT were documented. [^68^ Ga]Ga-PSMA-11 PET/CT findings were correlated with results of mpMRI and histopathology. A consensus of imaging, clinical and/or follow-up findings were used for determining the distant metastases, which were not verified by histopathology.

**Results:**

In the patient cohort who underwent RPE, [^68^ Ga]Ga-PSMA-11 PET/CT and mpMRI detected the IL in 86.5% and 98.1% of the patients, respectively. The median of the maximum standardized uptake value (SUVmax) in the intraprostatic IL was 12 (range, 4.7–67.8). Intraprostatic IL of the high-risk patients showed significantly higher SUVmax than those in patients with intermediate risk for distant metastases (*n* = 48; median: 17.84 vs. 8.77; *p* = 0.02).

In total 729 LN were removed by ePLND in 48 patients. The histopathology verified 26 pelvic lymph node metastases (pLNM) in 20.8% (10/48) of the patients, which have been correctly identified in 60% of the patients on [^68^ Ga]Ga-PSMA-11 PET/CT, and in 50% on mpMRI. All but one pLNM had a maximum diameter below 10 mm.

Bone metastases (BM) and distant LNM (dLNM) were found in 17.3% of the patients on [^68^ Ga]Ga-PSMA-11 PET/CT imaging. 39.0% of the [^68^ Ga]Ga-PSMA-11 PET-positive BM showed no suspicious morphological correlation on CT.

**Conclusion:**

[^68^ Ga]Ga-PSMA-11 PET/CT shows high diagnostic performance for N and M staging of patients with intermediate- and high-risk prostate cancer and seems to be superior to pelvic mpMRI in the detection of locoregional lymph node metastases. A significant correlation was found between SUVmax of the intraprostatic index lesion and risk stratification based on tPSA level and GS. The results of this study emphasize again on the role of metabolic molecular imaging using specific tracers in selected patients, leading to tailored therapy approach.

**Supplementary Information:**

The online version contains supplementary material available at 10.1007/s11307-021-01650-9.

## Introduction

Prostate cancer (PCa) is the second most common malignancy in men worldwide and the most diagnosed cancer in most European countries [1]. Therefore, the selection of the most suitable diagnostic pathway for accurate staging of the disease plays a pivotal role in management of the PCa patients guiding to the appropriate treatment approach [2]. To date, there is no recommendation on the use of Gallium-labeled prostate-specific membrane antigen positron emission tomography-computed tomography ([^68^ Ga]Ga-PSMA-11 PET/CT) imaging for primary staging of PCa in the current EAU-ESTRO-SIOG Guidelines [3]. However, multiparametric magnetic resonance imaging (mpMRI) is recommended for local staging in patients with intermediate- and high-risk PCa. For the assessment of distant metastases, cross-sectional abdomen-pelvic imaging by computed tomography (CT) and/or bone scan are recommended [3, 4]. Prostate-specific membrane antigen (PSMA) is a type II transmembrane protein that is expressed on the cell surface of prostatic cells, but also in the neovasculature of other malignant neoplasms [5]. It is overexpressed up to 1000 times in PCa cells compared to normal prostate epithelial cells [6]. This biological characteristics enables excellent depiction of the PCa cells by [^68^ Ga]Ga-PSMA-11 PET/CT imaging [7, 8]. It has also been shown that there is a positive correlation between PSMA expression and Gleason score (GS). Moreover, a high PSMA expression is significantly associated with a higher risk of disease recurrence following curative surgery [9]. In recent years, several studies assessed the potential of radiolabeled-PSMA-11 PET/CT in PCa; however, the main focus was on patients with biochemical recurrence [10–12]. There are far less studies comparing the diagnostic value between [^68^ Ga]Ga-PSMA-11 PET/CT and mpMRI in the staging setting, particularly in the assessment of pelvic lymph nodes. In addition, most of previous investigations presented retrospective data [13–18].

To the best of our knowledge, there are only few studies in this concern with prospective design [19].

Considering the limitations addressed in the previous studies, mainly retrospective design without histopathologic correlations and clear standard of proof, we performed this prospective clinical trial to assess the value of [^68^ Ga]Ga-PSMA-11 PET/CT in pre-operative N and M staging of intermediate- and high-risk PCa patients compared to pelvic multi-parametric magnetic resonance imaging (mpMRI) and histopathological findings. Furthermore, we investigated the agreement between [^68^ Ga]Ga-PSMA-11 PET/CT and mpMRI in the detection of the intraprostatic index lesion (IL) and assessed the impact of clinical biomarkers on [^68^ Ga]Ga-PSMA-11 PET-positivity.

## Patients and Methods

This prospective single-center study was performed in accordance with the principles of the 1964 Declaration of Helsinki and its later amendments or comparable standards and approved by the routing ethics committee of the province with trial number of “415-E/2146/4–2017” and was registered in the European Clinical Trials Database (EudraCT number: 2020–000,771-20). Written informed consent was obtained from all individuals participated in the study according to the guidelines of the Ethics Committee.

All included patients were scheduled for [^68^ Ga]Ga-PSMA-11 PET/CT, pelvic mpMRI, and radical prostatectomy (RPE). Trigger PSA (tPSA), Gleason score (GS), and National Comprehensive Cancer Network (NCCN) risk stratification based on biopsy were recorded. The final diagnosis was principally based on histopathological sections from RPE. In case of lack of histopathology, undetermined findings, or disagreement between two readers, the final interpretation has been defined by an interdisciplinary consensus using other imaging modalities and clinical findings.

### Patients

Overall, 81 consecutive patients (mean age 64.5 years, 45.3–79.1/mean tPSA 15.4 ng/ml; range, 4.1–94.0) have been prospectively included between September 2017 and January 2020. The inclusion criteria were transrectal ultrasound (TRUS)-guided biopsy-proven prostate cancer, without any clinical evidence of metastases and a median Eastern Co-operative Oncology Group (ECOG) performance status of zero, who were principally scheduled for RPE. The criteria for patient selection are shown in Table 1.

**Table 1.** Patients per modality and characteristics of patients who underwent RPE

		NCCN risk classification
Included patients (*n*)	**81**	Intermediate 24 (29.6%) High 57 (70.4%)
Patients with PET/CT and mpMRI (*n*)	71	Intermediate 22 (31.0%) High 49 (69.0%)
Patients with RPE + ePLND (*n*)	54	Intermediate 24 (44.4%) High 30 (55.6%)
Patients with PET/CT, mpMRI and RPE + ePLND (*n*)	52	Intermediate 23 (44. 2%) High 29 (55.8%)
Age, median (range)	65.9 (45.3–79.1)	
tPSA median (range)	10.04 ng/ml (4.1–94)	
	Characteristics	Value
Gleason score RPE, *n* (%)	6	1 (1.9%)
	7a	24 (44.4%)
	7b	8 (14.8%)
	8	6 (11.1%)
	9	15 (27.8%)
pT stage, *n* (%)	T2c	30 (55.5%)
	T3a	11 (20.4%)
	T3b	11 (20.4%)
	T4	2 (3.7%)

### Image Acquisition

#### [^68^ Ga]Ga-PSMA-11 PET/CT

Imaging was performed systematically according to the study protocol from the base of skull to the proximal femur (PET-Position 2 min 40 s, 2 mm slices) using two different PET/CT scanners (Philips Ingenuity TF, Amsterdam/the Netherlands, and Siemens Biograph mCT, Erlangen/Germany). Mean interval between [^68^ Ga]Ga-PSMA-11 application and the start of the acquisition was 60 min (SD ± 14.4 min). The mean injected activity per kg/bodyweight was 2.15 MBq. A non-contrast-enhanced low-dose CT scan (Siemens: Care Dose 4D, Care kV, slice thickness 1.2 mm and pitch 1.5; Philips: 100 kV, 33 mAs, slice thickness 1.5 mm and pitch 0.8) was performed for attenuation correction and localization. Slices measuring 3 mm were reconstructed using iDose mode level 3 (Philips Ingenuity TF), respectively, SAFIRE level 3 (Siemens Biograph). Both PET/CT scanners are EARL/EANM (EANM Research Ltd/European Association of Nuclear Medicine) accredited. Therefore, we assume that the performance of both scanners is almost similar. PSMA-11 (Glu-NH-CO–NH-Lys(Ahx)-HBED-CC) form ABX advanced biochemical compounds (Radeberg, Germany) was synthesized using the SCINTOMICS GRP^®^ module. The used procedure follows the manufacturer’s information (version 2.0). Several quality checks were performed including thin-layer chromatography (TLC) and high-performance liquid chromatography (HPLC) to guarantee a radiochemical purity > 95 %.

#### Multiparametric MRI

The mpMRI images were performed covering the pelvis using a 3 Tesla MRI (Achieva, Philips Medical Systems, Best/the Netherlands) with an anterior coil. Patient position was “feet first,” and a pelvis compression belt was used. The MRI exam was performed and interpreted in accordance with the current Prostate Imaging Reporting and Data System version 2 guidelines (PI-RADS v 2.0) [20]. T2-weighted turbo spin-echo (3 mm sagittal, transversal and coronary slices) and transversal diffusion-weighted (DWI) sequences, including high b-value images (0/50/1000/2000s/mm^2^), were acquired. After injection of 1 ml per bodyweight contrast media gadobutrol (Gadovist, Bayer Healthcare, Germany) dynamic T1-weighted fast field echo and post-contrast T1-weighted images were obtained, the FOV was focused on the prostate. In addition, DWI images (b-value images 0/800/1200 s/mm^2^) and transversal post-contrast DIXON-W T1-weighted images with a larger FOV for pelvic lymph node evaluation and covering the entire pelvis were performed.

### Image interpretation

#### [^68^ Ga]Ga-PSMA-11 PET/CT

Reporting was done systematically using a segmentation of the prostate in six segments (left and right — base, mid, apex) for [^68^ Ga]Ga-PSMA-11 PET/CT. An increased, non-physiologic uptake more than background activity in each organ (e.g., prostate, bone) or more than blood pool (e.g., lymph nodes) was considered as abnormal. Images were evaluated by two board-certified nuclear medicine physicians (L.R. and G.S-Z.) who were aware of all available clinical data, but blinded to the localization of the primary tumor as known from the transrectal ultrasound (TRUS)-guided biopsy. Lesion-based mean and maximum standardized uptake value (SUVmean/max) were measured using a 40 % threshold of maximum activity in the 3D volume of interest (VOI) by using the commercially available image viewing software IntelliSpace Portal (Koninklijke Philips N.V., 2015). The lesion with the highest SUVmax was defined as “index lesion” (IL). Number and position of lymph nodes and bone lesions with abnormal increased uptake have been documented.

#### Multiparametric MRI

All images were evaluated by an experienced board-certified uro-radiologist (M.M.), using the PI-RADS v2-scoring system [20], who was aware of all available clinical data, but was blinded to the location of the primary tumor as known from the TRUS biopsy and the PET/CT images. A second experienced uro-radiologist (R.F.) has supervised the reading especially in undetermined cases. The lesion with the highest PIRADS classification was defined as IL. If there were more lesions with the same PIRADS classification, the lesion with the lager diameter was defined as IL.

### Correlation of Intraprostatic Imaging Findings with Histopathology

For an inter-modality comparison of the anatomic location of the intraprostatic lesions, the 6-segment model (left and right — base, mid, apex) was used for each modality. [^68^ Ga]Ga-PSMA-11 PET/CT results included the anatomic location of the lesions and SUVmax values. mpMRI results included the anatomic location of the lesions, PI-RADS v2 score, and TNM classification. Histopathological results included the anatomic location of the lesions, GS based on prostatectomy, residual tumor, and TNM classification, as well as number, size, and location of malignant lymph nodes (LN). In addition, GS based on biopsy and tPSA values at the date of the [^68^ Ga]Ga-PSMA-11 PET/CT examination were collected. RPE specimen were routinely grossly dissected with clearly defined localization and diagnosed including the GS according to the College of American Pathologists (CAP) guidelines. Prostate tissue was evaluated histologically, and hematoxylin–eosin stained tissue sections were analyzed. For the categorization of imaging findings, a histological tumor burden of more than 60 % of malignant cells in the respective section in one of the six segments was defined as positive. Histopathological evaluation was done by an experienced board-certified pathologist (C.H–K.).

### Statistical Analysis

Data were crosschecked for consistency and normality. Data were visually inspected for invalid values and outliers by using probability plots and Kolmogorov–Smirnov test was applied to test for normality. In case of non-normal data, Kruskal–Wallis ANOVA and Mann–Whitney *U* test were applied to compare medians among various independent groups, and Spearman correlation coefficients were used to analyze the correlations between various continuously distributed variables such as SUVmax and tPSA level. The correlation between SUVmax and GS based on biopsy and/or histopathology after prostatectomy, respectively, was assessed by Kruskal–Wallis ANOVA. The differences in median SUVmax values between tumors with GS ≤ 7b and those with GS of ≥ 8, between tumors with T-stage ≤ pT2c and those with ≥ pT3a, and between tumors with UICC stadium ≤ 2 and ≥ 3, respectively, were calculated by Mann–Whitney *U* test. Sensitivity, specificity and accuracy with 95 % CI (Pearson-Clopper) were computed when appropriate. All reported tests were two-sided, and *p* values < 0.05 were considered as statistically significant. All statistical analyses in this report were performed by use of NCSS (NCSS 10, NCSS, LLC. Kaysville, UT), STATISTICA 13 (Hill, T. & Lewicki, P. Statistics: Methods and Applications. StatSoft, Tulsa, OK), and PASW 24 (IBM SPSS Statistics for Windows, Version 21.0., Armonk, NY).

## Results

Overall, 81 PCa patients were included for primary N and M staging in this study and underwent [^68^ Ga]Ga-PSMA-11 PET/CT. However, mpMRI and postoperative histopathologic findings were available in 52 cases. The median interval between two imaging modalities was 2 days (range, 0–16 days). According to National Comprehensive Cancer Network (NCCN) criteria [21], 29.6 % (24/81) patients were classified as intermediate risk and 70.4 % (57/81) as high risk for extra-glandular metastases. Among the 52 patients who underwent [^68^ Ga]Ga-PSMA-11 PET/CT, mpMRI and RPE, 44.2 % (23/52) were classified as intermediate risk and 55.8 % (29/52) as high risk. Patient’s characteristics and details of performed imaging modalities are shown in Table 1. The reasons for missing clinical or imaging data are shown in an additional table (see ESM 1). Statistical data and correlation analysis are summarized in the additional table 2 (see ESM 2).

### Primary Tumor (T-Stage)

[^68^ Ga]Ga-PSMA-11 PET/CT detected the IL in 88.9 % (72/81) of the patients, whereas in 11.1 % (9/81) of the patients, the primary tumor was not clearly distinguishable from normal prostate tissue. These nine patients showed a median tPSA of 10.1 ng/ml (range, 5.30–12.3 ng/ml). In one of these patients, [^68^ Ga]Ga-PSMA-11 PET/CT detected distant LNM (supraclavicular/tPSA 11.99 ng/ml/GS 7b). Eight out of these nine [^68^ Ga]Ga-PSMA-11 PET-negative patients underwent mpMRI. All eight patients were classified as cT-stage ≥ T2a. In the patient cohort with mpMRI and RPE, [^68^ Ga]Ga-PSMA-11 PET/CT and mpMRI detected the IL in 86.5 % (45/52) and 98.1 % (51/52) of the patients, respectively. The sensitivity for the detection of the IL was 88.9 % (CI 81–94 %) and 98.6 % (CI 93–99.9 %) for [^68^ Ga]Ga-PSMA-11 PET/CT and mpMRI, respectively.

**Fig. 1.**
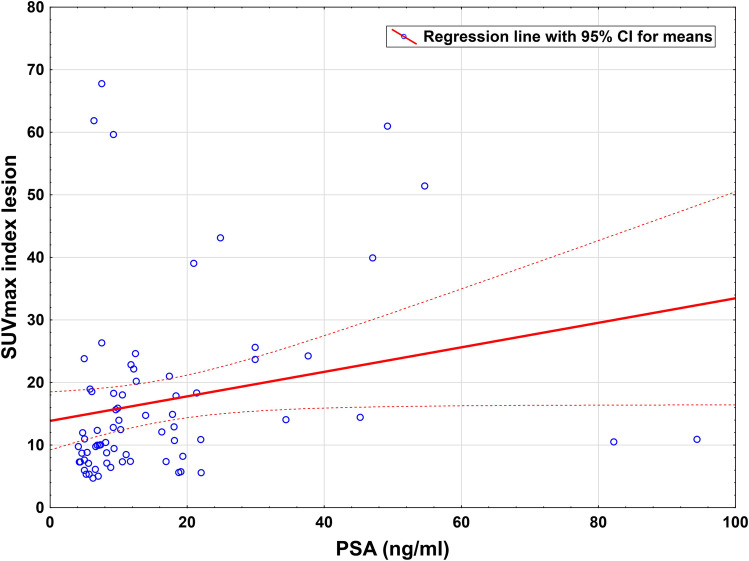
Comparison of tPSA levels and SUVmax. *r* (Spearman) = 0.38; *p* < 0.001.

The median SUVmax in the IL was 12 (range, 4.7–67.8). Only a weak correlation (*n* = 72, *r*_s_ = 0.38, *p* = 0.001) was found between the SUVmax and tPSA level (Fig. 1). The difference in the SUVmax according to the particular GS was not statistically significant, neither for GS based on biopsy (*n* = 70, *p* = 0.3), nor for GS-based histopathological findings prostatectomy specimens (*n* = 48, *p* = 0.39). The comparison of SUVmax values of the IL of different GS subgroups are shown in the supplemental Fig. 2 (see ESM 3).

**Fig. 2.**
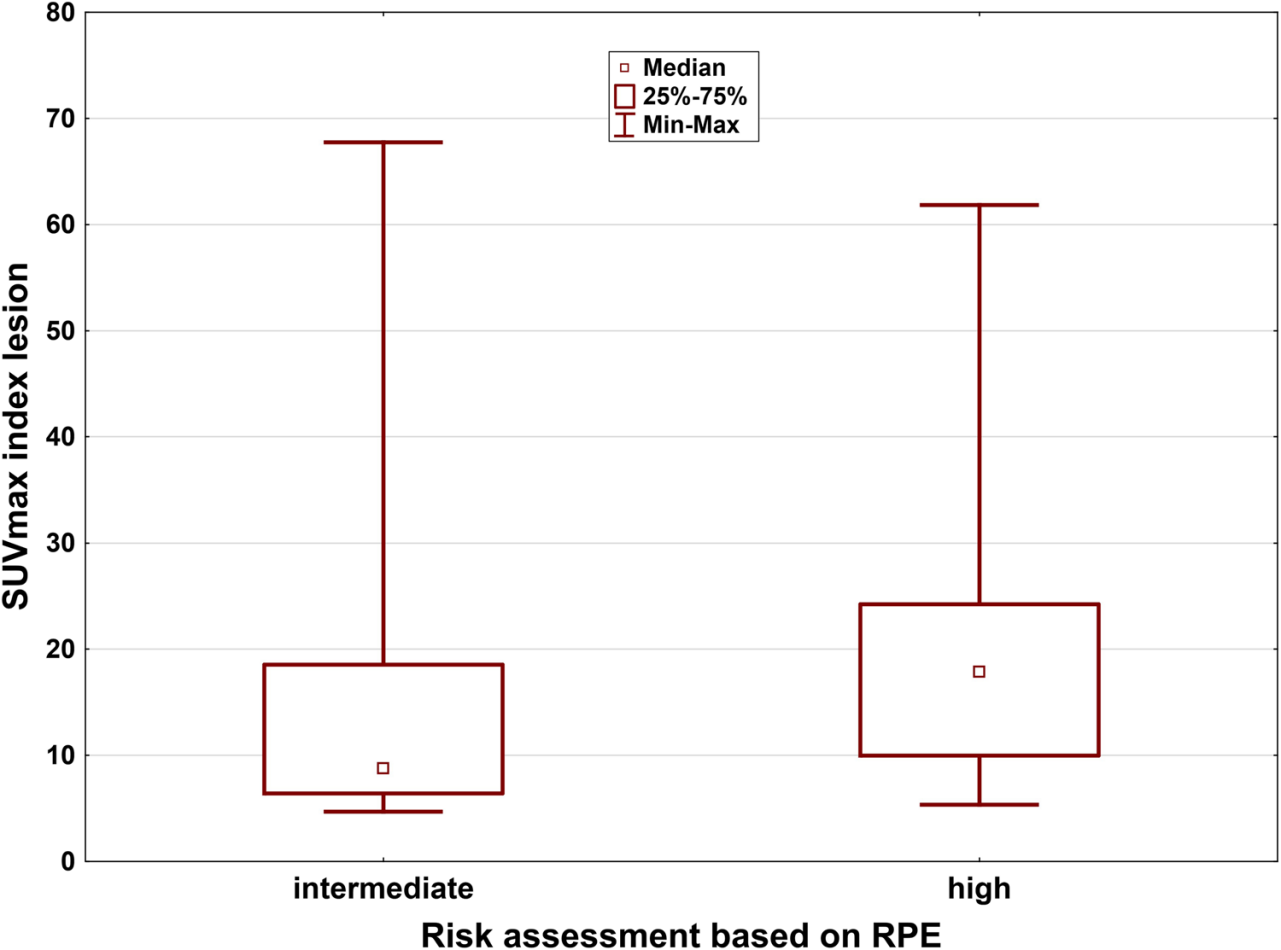
SUVmax and risk assessment based on GS in RPE specimen and tPSA value (*p* = 0.02).

A significant difference was found in maximum SUV levels of the intraprostatic index lesion between intermediate compared to high-risk patients based on tPSA and GS obtained from histopathology (*n* = 48, median: 8.77 vs. 17.84; 25–75 % quartile: 6.41–18.55 vs. 9.98–24.24; Mann–Whitney *U* test: *p* = 0.02) (Fig. 2). However, there were no statistically significant differences in median SUVmax between tumors with T-stage pT2c and ≥ pT3a (*n* = 48, *p* = 0.098) and between tumors with Union Internationale Contre le Cancer (UICC) stadium ≤ 2 and ≥ 3 (*n* = 48, *p* = 0.056), respectively. Comparison of median SUVmax values in patients with T-stage pT2c and ≥ pT3a is shown in the supplemental Fig. 3 (see ESM 4).

**Fig. 3.**
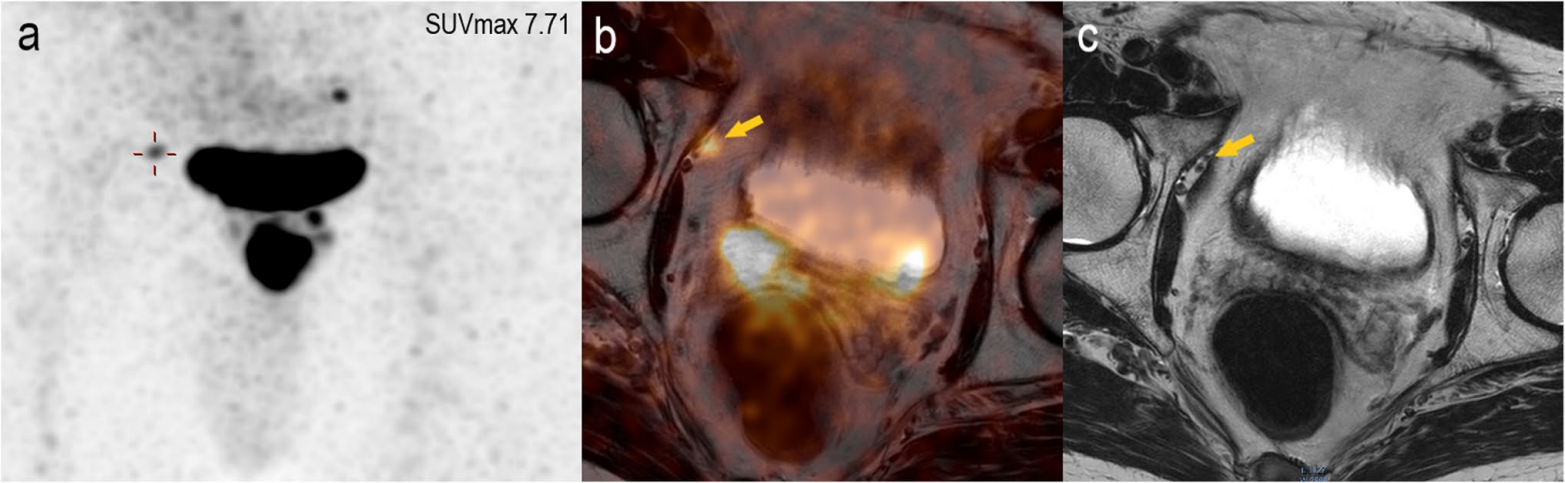
The potential of [^68^ Ga]Ga-PSMA-11 PET in the detection of small LN clearly superior to mpMRI. A 71-year-old high-risk prostate cancer patient, Gleason score 6 and tPSA of 20.96 ng/ml showed suspicious uptake in a pelvic LN in the maximum intensity projection image (**a**) and the fused PET/CT image (**b**). However, it did not fulfill the criteria for malignancy on T2-weighted MR imaging (**c**).

### LNM (N-Stage)

In total 729 LN were removed by ePLND in 48 patients, and in 20.8 % (10/48) of the patients, malignant LN were detected in the histologic evaluation (in total 26 malignant LN). Out of these 10 patients, 60 % (6/10) showed LN metastases on [^68^ Ga]Ga-PSMA-11 PET/CT, while it was 50 % (5/10) on mpMRI considering the same FOV. The sensitivity, specificity, and accuracy for the detection of pelvic LNM were 60 %, 91 %, and 83 % for [^68^ Ga]Ga-PSMA-11 PET/CT and 50 %, 97 %, and 87 % for mpMRI, respectively. In 5 patients, both imaging modalities were able to detect pelvic LNM. Out of these 26 LNM, 61.5 % (16/26) were positive on [^68^ Ga]Ga-PSMA-11 PET/CT and 38.5 % (10/26) on mpMRI, respectively. Four histologically verified LNM were detected neither by [^68^ Ga]Ga -PSMA-11 PET/CT nor by mpMRI. In 3 patients with [^68^ Ga]Ga-PSMA-11 PET-positive LN, no cancerous tissue was reported in the dissected LN on the histopathological examinations. All but one [^68^ Ga]Ga-PSMA-11 PET- negative LNM removed measured less than 10 mm in size with a median size of 2 mm (range, 0.6–13 mm), while the median size of the [^68^ Ga]Ga-PSMA-11 PET-positive LNM was 6 mm (range, 0.6–18 mm).

### Distant Metastases (M-Stage)

Bone metastases (BM) were found in 14.8 % (12/81) and 9.9 % (7/71) of the patients on [^68^ Ga]Ga-PSMA-11 PET and pelvic mpMRI, respectively. In 6 patients, both imaging modalities were able to detect BM in the pelvic region. In one case, the MRI showed an unclear osseous lesion with a strong contrast enhancement. In total 41 PET-positive bone lesions were seen. However, 26.8 % (11/41) of the [^68^ Ga]Ga-PSMA-11 PET-positive BM in 58.3 % (7/12) of these patients were outside the MRI-FOV (ribs, scapula, vertebral bodies). Out of them, 5 patients had oligometastasis. Only 18.2 % (2/11) of the [^68^ Ga]Ga-PSMA-11 PET-positive distant bone lesions in 2 patients showed appreciable morphological changes on CT suggestive of metastases.

No suspicious morphological changes were seen on CT in 39.0 % (16/41) of the [^68^ Ga]Ga-PSMA-11 PET-positive BM. [^68^ Ga]Ga-PSMA-11 PET/CT detected early bone marrow involvement in 41.7 % (5/12) of the patients (intermediate risk *n* = 2, high risk *n* = 3), before morphological changes appeared on CT. There was a significant correlation between distant metastases detected by [^68^ Ga]Ga-PSMA-11 PET/CT and mpMRI-based T-stage (*n* = 11, *p* = 0.0001) and tPSA (*n* = 14, *p* = 0.004). However, no remarkable correlation was found between distant metastases and biopsy-based GS (*n* = 14, *p* = 0.65).

Overall, [^68^ Ga]Ga-PSMA-11 PET/CT was able to detect distant metastases in 17.3 % (14/81) of the patients.

## Discussion

[^68^ Ga]Ga-PSMA-11 PET/CT in assessment of PCa patients was the focus of a large number of publications in the last years. However, there are still limited prospective clinical trials assessing the [^68^ Ga]Ga-PSMA-11 PET/CT in primary staging of PCa in an adequate patient’s population comparing to standard of care mpMRI. In this prospective study, we evaluated the value of [^68^ Ga]Ga-PSMA-11 PET/CT in pre-operative staging of intermediate- and high-risk PCa patients compared to pelvic mpMRI and histopathological findings.

**Fig. 4.**
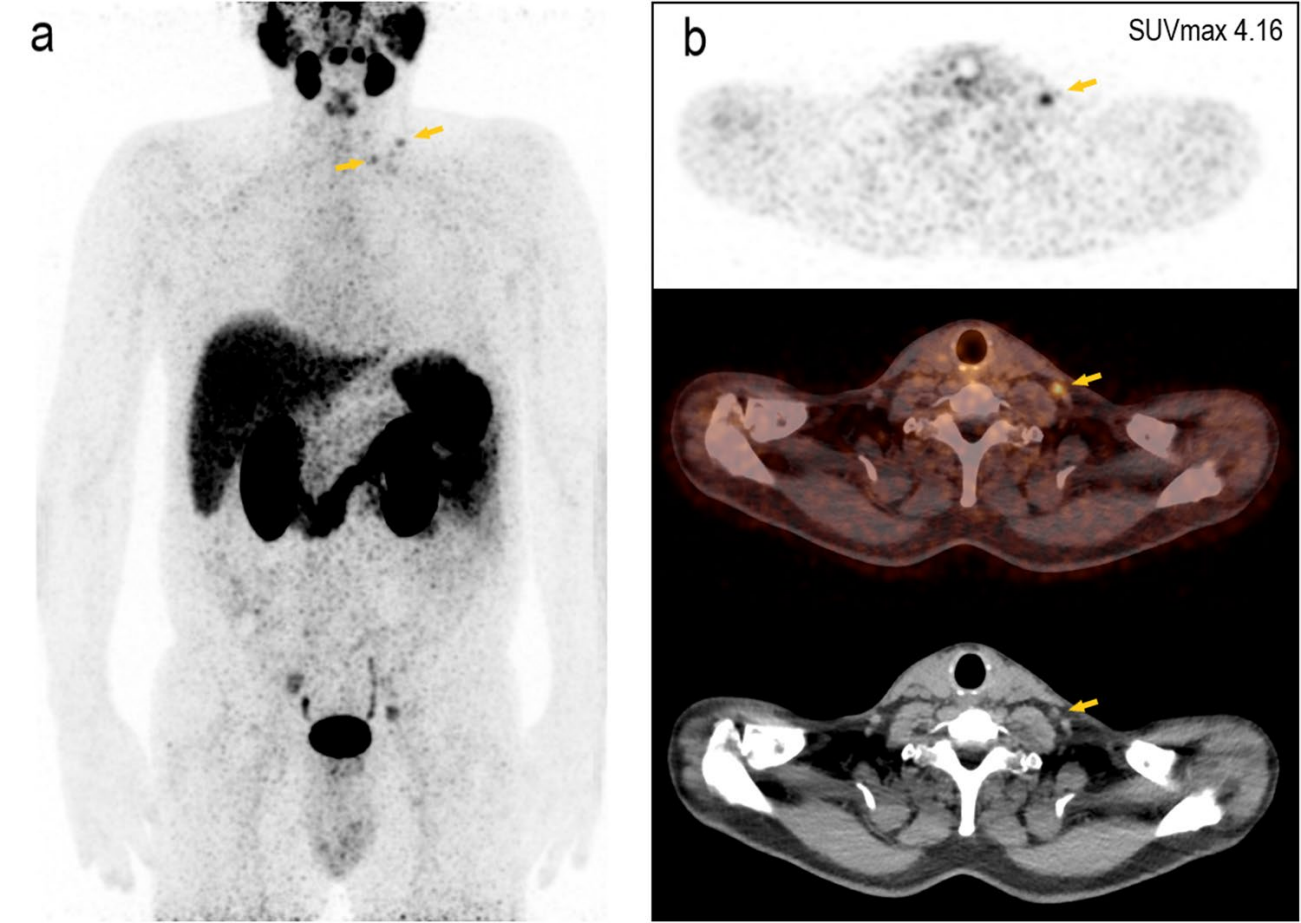
The potential of [^68^ Ga]Ga-PSMA-11 PET in the detection of distant LNM. A 59-year-old high-risk prostate cancer patient, Gleason score 7b, and tPSA of 11.99 ng/ml showed suspicious uptake in LN supraclavicular left. **a** Maximum intensity projection image; **b** PET image, fused PET/CT image, and CT image alone.

**Fig. 5.**
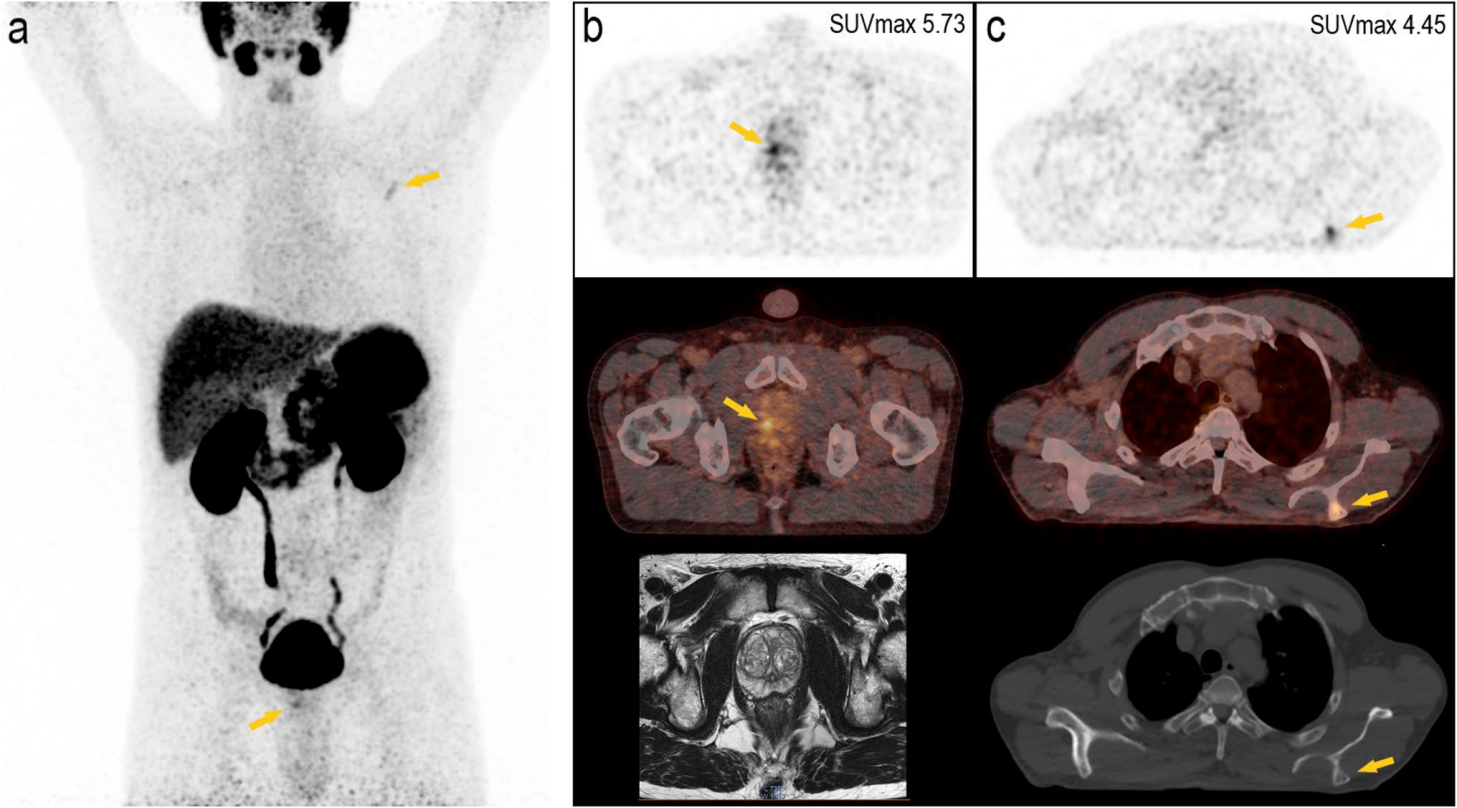
The additive value of [^68^ Ga]Ga-PSMA-11 PET/CT in the detection of distant bone metastases. A 58-year-old intermediate-risk prostate cancer patient, Gleason score 7a, and tPSA of 19.08 ng/ml showed suspicious focal uptake on the left spine of the scapula without morphological changes on CT. **a** Maximum intensity projection image; **b** index lesion on PET imaging, index lesion on fused PET/CT imaging, and T2-weighted MRI (PIRADS 3); **c** bone lesion on PET imaging, bone lesion on fused PET/CT imaging, and CT image in bone window.

The results of this clinical trial showed a high diagnostic performance of [^68^ Ga]Ga-PSMA-11 PET/CT imaging in primary staging of biopsy-proven PCa, particularly in accurate detection of lymph nodes and distant metastases (Figs. 4 and 5), which led to tailored treatment strategy for each individual and optimal management of the disease. In the detection of primary intraprostatic PCa, mpMRI showed higher sensitivity of 98.6 % comparing to 88.9 % for [^68^ Ga]Ga-PSMA-11 PET/CT. Although, some studies found no difference between [^68^ Ga]Ga-PSMA-11 PET/CT and MRI in the detection of intraprostatic index lesions; however, similar to our findings, it is generally accepted that mpMRI is superior to [^68^ Ga]Ga-PSMA PET/CT in the assessment of the primary tumor (T-stage) [3, 22, 23].

In the detection of pelvic LNM, [^68^ Ga]Ga-PSMA-11 PET/CT showed higher sensitivity comparing to mpMRI (Fig. 3). In a previous study, Franklin et al. evaluated the predictive value of mpMRI and [^68^ Ga]Ga-PSMA PET/CT for pathological outcomes at RPE and PLND. The authors reported that preoperative [^68^ Ga]Ga-PSMA PET/CT was more sensitive in identifying histologically verified pelvic LNM than mpMRI. However, [^68^ Ga]Ga-PSMA PET/CT was negative in 32.8 % (20/61) of the high-risk patients with microscopic LNM [24]. Our results are comparable with these reported findings. In 40 % (4/10) of the patients, the micro-metastatic lymph nodes (< 3 mm) were not detectable by [^68^ Ga]Ga-PSMA-11 PET/CT. All but one [^68^ Ga]Ga-PSMA-11 PET-negative LNM removed measured less than 10 mm in size with a median size of 2 mm, while the median size of the [^68^ Ga]Ga-PSMA-11 PET-positive LNM was 6 mm. These findings are in accordance with the data reported in a recent study showing the median size of undetected LNM of 4.3 mm (range 1.0–10.8 mm) on [^68^ Ga]Ga-PSMA-11 PET/CT [25].

Another recent study by Esen et al. evaluated the accuracy of preoperative [^68^ Ga]Ga-PSMA-11 PET/CT in the detection of LNM in intermediate- or high-risk PCa patients. The authors reported a per-patient sensitivity, specificity, and accuracy of 53.3 %, 98.8 %, and 91.7 % in the detection of LNM, respectively [17]. The minor differences of the results may be related to the number of included patients with different risk stratifications, surgical approaches, and mean number of dissected lymph nodes.

In addition, [^68^ Ga]Ga-PSMA-11 PET/CT changed the therapeutic management of the disease in about 20 % of the patients, similar to the previous reported findings which showed superior accuracy of [^68^ Ga]Ga-PSMA PET/CT when compared to the combined findings of CT and bone scanning (Fig. 5) [26–28]. In our study, only 18.2 % (2/11) of the [^68^ Ga]Ga-PSMA-11 PET-positive distant bone lesions showed appreciable morphological changes on CT suggestive of metastases.

PSA value and GS are well-accepted markers for risk stratification guiding the use of further diagnostic tools. Notably, our results suggest that the median tPSA alone seems not to be an accurate marker for selecting patients who may benefit from [^68^ Ga]Ga-PSMA-11 PET/CT, since nine patients with tPSA levels below 10 ng/ml had already LN or bone metastases. Nevertheless, our data showed a significant correlation between occurrence of distant metastases and mpMRI-based T-stage and tPSA, which could serve as clinical predictors of bone metastases on [^68^ Ga]Ga-PSMA-11 PET/CT. Also, a previous study reported that extra-prostatic disease is predictive of a poor surgical response (SR) to RPE and [^68^ Ga]Ga-PSMA-11 PET/CT had a superior predictive value for SR than GS, tPSA, or T-stage [29].

However, due to limited sample size, creating a prediction model for analysis of risk-stratification of extra-prostatic metastases was not possible. Recent studies reported various results concerning the correlation between SUVmax and PSA value [30–32]. We found a significant correlation between tPSA and SUVmax in the IL confirming the findings of Cytawa et al. [32] who reported weak correlation between SUVmax of primary tumors and tPSA levels. In addition, we found no association between SUVmax and GS. Using a risk assessment including histopathology-based GS and tPSA value, we found a significant difference between SUVmax levels in the IL in intermediate compared to high-risk patients. Generally, histopathology relies on a threshold for tumor cell invasion in the respective segment. In our study, a 60 % threshold was used to outline the primary tumor. Few studies reported the respective thresholds applied, and this might therefore explain controversial results concerning the relation between the intensity of [^68^ Ga]Ga-PSMA-11 uptake in the primary tumor and GS (rPearson = 0.66; *p* < 0.001) [31, 33].

This study has some limitations. Bone metastases and distant LNM could not be histologically verified. We employed a CT protocol without contrast agent, which reduces the sensitivity in detecting lymph nodes based on the CT criteria only. However, all but one LNM were smaller than 10 mm. Although we have tried to mark dissected lymph nodes and correlate them with corresponding imaging findings, a 1:1 verification was not possible. This was mainly due to the reason that only the region of resected lymph nodes will be documented by the urologists. Thus, they were not able to mark each single LN that has been detected on imaging modalities. The comparison between [^68^ Ga]Ga-PSMA-11 PET/CT and mpMRI performance was restricted to the pelvic region due to the limited mpMRI-FOV.

## Conclusions

[^68^ Ga]Ga-PSMA-11 PET/CT shows high diagnostic performance for N and M staging of patients with intermediate- and high-risk prostate cancer and seems to be superior to pelvic mpMRI in the detection of locoregional lymph node metastases. A significant correlation was found between SUVmax of the intraprostatic index lesion and risk stratification based on tPSA level and GS. The results of this study emphasize again on the role of metabolic molecular imaging using specific tracers in selected patients, leading to tailored therapy approach.

## Supplementary Information

Below is the link to the electronic supplementary material.

Supplementary file1 (DOCX 14 KB)

Supplementary file2 (DOCX 24 KB)

Supplementary file3 (DOCX 231 KB)

Supplementary file4 (DOCX 115 KB)

## Data Availability

Trial number: 415-E/2146/4–2017; EudraCT number: 2020–000,771-20.
