# Supplementary material for: Incremental Impact of [68 Ga]Ga-PSMA-11 PET/CT in Primary N and M Staging of Prostate Cancer Prior to Curative-Intent Surgery: a Prospective Clinical Trial in Comparison with mpMRI
Source: Mol Imaging Biol. 2021 Sep 14;24(1):50–9. doi: 10.1007/s11307-021-01650-9 (PMC8760214; doi:10.1007/s11307-021-01650-9)
Supplement: Supplementary file 1 — Supplementary file1 (DOCX 14 KB) [file 11307_2021_1650_MOESM1_ESM.docx]

**Supplement 1** Reasons for not performing mpMRI or RPE

| Reasons for not performing **mpMRI** | - patients did not tolerate the examination (n=5) - patients did not show up (n=5). |
| --- | --- |
| Reasons for not performing **RPE** | - detection of distant metastases (n=6) - poor clinical condition (n=2) - patients chose radiation therapy (n=7) - patients chose active surveillance (n=1) |
| Missing clinical and imaging follow-ups | n=11 |
