# Supplementary material for: Incremental Impact of [68 Ga]Ga-PSMA-11 PET/CT in Primary N and M Staging of Prostate Cancer Prior to Curative-Intent Surgery: a Prospective Clinical Trial in Comparison with mpMRI
Source: Mol Imaging Biol. 2021 Sep 14;24(1):50–9. doi: 10.1007/s11307-021-01650-9 (PMC8760214; doi:10.1007/s11307-021-01650-9)
Supplement: Supplementary file 2 — Supplementary file2 (DOCX 24 KB) [file 11307_2021_1650_MOESM2_ESM.docx]

**Table 2**
Statistical data and correlation analysis

|  | mpMRI | [^68^Ga]Ga-PSMA-11 PET/CT |
| --- | --- | --- |
| Index Lesion (n=52) |  | |
| Sensitivity | 98.6% | 88.9% |
| Histological pelvic LNM (n=26) |  | |
| detect pelvic LNM | 10/26 | 16/26 |
| Sensitivity (per-patient) | 50% | 60% |
| Specificity (per-patient) | 97% | 91% |
| Accuracy (per-patient) | 87% | 83% |
| BM |  | |
| Patients with BM | 7/71 | 12/81 |
|  | | |
| SUVmax and tPSA (n=72) |  | |
| SUVmax: median (range) | 12 (4.7–67.8) | |
| r_Spearman_ (p-value) | 0.38 (0.001) | |
|  | | |
| SUVmax and GS |  | |
| GS biopsy: p-value (n) | 0.30 (70) | |
| GS RPE: p-value (n) | 0.39 (48) | |
|  | | |
| SUVmax and risk assessment (n=48) |  | |
| intermediate: SUVmax median (range) | 8.77 (4.71-67.76) | |
| high: SUVmax median (range) | 17.84 (5.34-61.68) | |
| p-value (Mann-Whitney U test) | 0.02 | |
|  | | |
| PSMA PET/CT based M-Stage and mpMRI-based T-Stage (n=11) |  | |
| p-value (Pearson Chi-square) | 0.0001 | |
|  | | |
| PSMA PET/CT based M-Stage and tPSA (n=14) |  | |
| p-value (logistic regression) | 0.004 | |
