# Supplementary material for: Incremental Impact of [68 Ga]Ga-PSMA-11 PET/CT in Primary N and M Staging of Prostate Cancer Prior to Curative-Intent Surgery: a Prospective Clinical Trial in Comparison with mpMRI
Source: Mol Imaging Biol. 2021 Sep 14;24(1):50–9. doi: 10.1007/s11307-021-01650-9 (PMC8760214; doi:10.1007/s11307-021-01650-9)
Supplement: Supplementary file 3 — Supplementary file3 (DOCX 231 KB) [file 11307_2021_1650_MOESM3_ESM.docx]

**a**


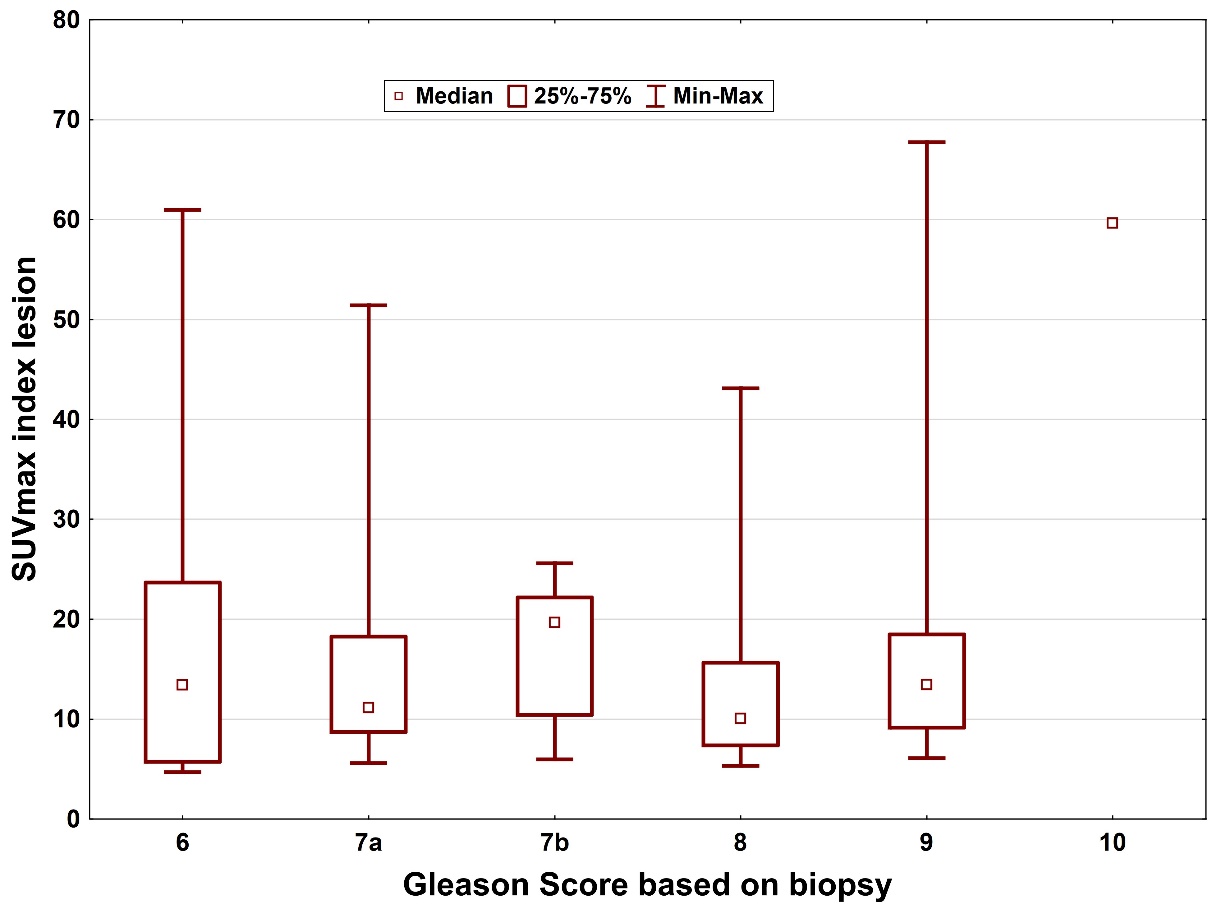


**b**


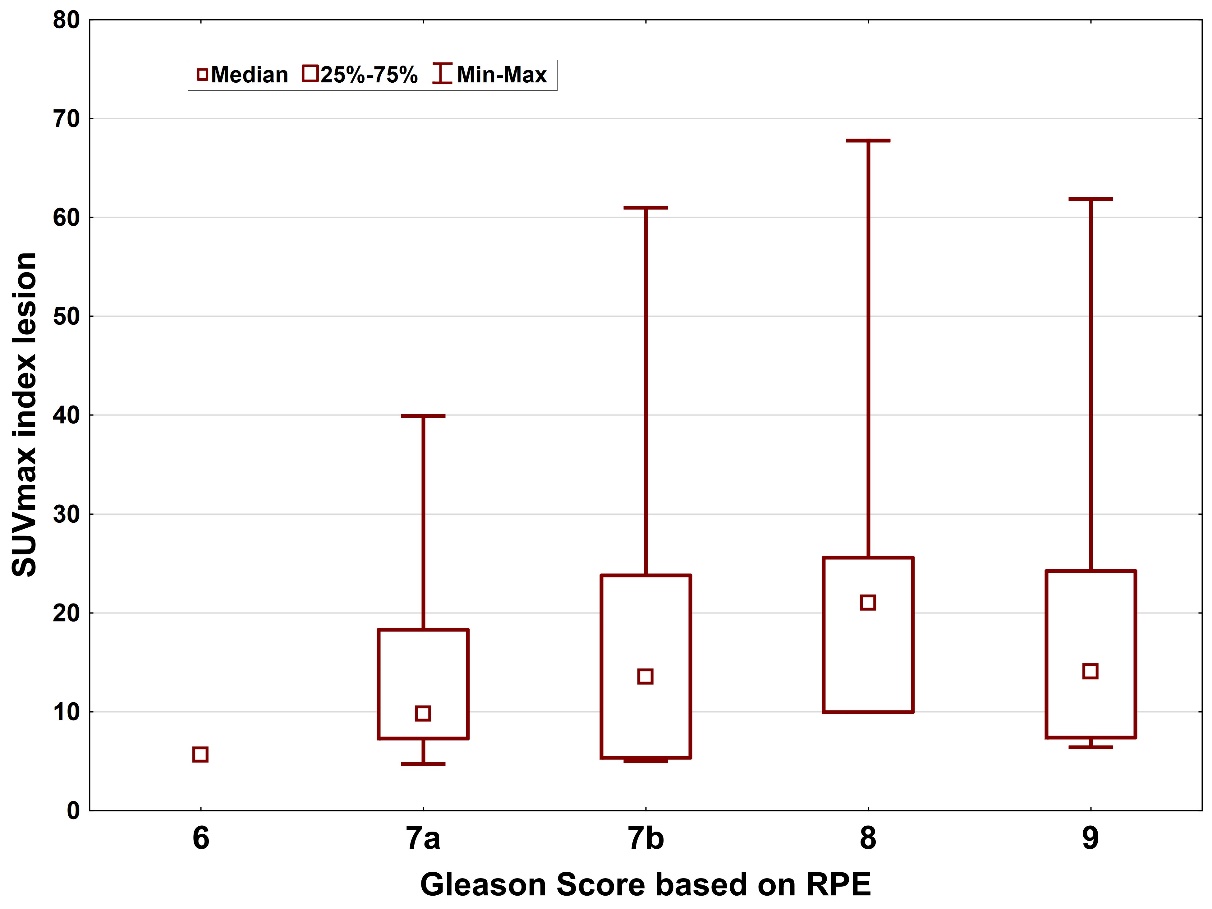
**supplementary material 2.** Comparison of SUVmax values of the index lesion (IL) of different Gleason score subgroups (a: based on biopsy and b: on histopathology of RPE specimen). Box plots demonstrate that there is no statistically correlation between SUVmax in the IL and the GS.
