# Supplementary material for: Incremental Impact of [68 Ga]Ga-PSMA-11 PET/CT in Primary N and M Staging of Prostate Cancer Prior to Curative-Intent Surgery: a Prospective Clinical Trial in Comparison with mpMRI
Source: Mol Imaging Biol. 2021 Sep 14;24(1):50–9. doi: 10.1007/s11307-021-01650-9 (PMC8760214; doi:10.1007/s11307-021-01650-9)
Supplement: Supplementary file 4 — Supplementary file4 (DOCX 115 KB) [file 11307_2021_1650_MOESM4_ESM.docx]

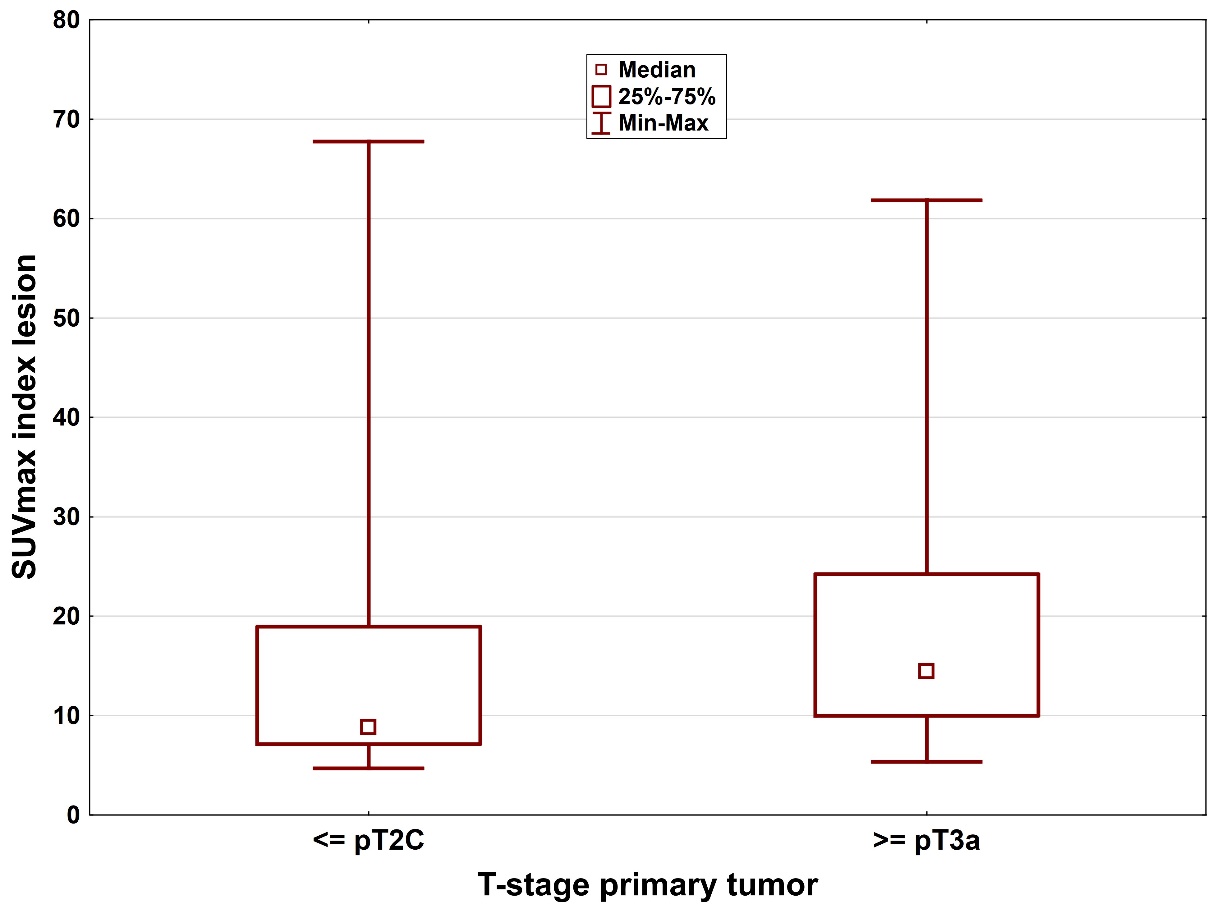


**supplementary material 3.** Comparison of median SUVmax values in patients with T-stage pT2c and ≥ pT3a. There was no statistically significant difference in median SUVmax between tumors with T-stage pT2c and ≥ pT3a (p= 0.098).
